# Endothelium in Spots – High-Content Imaging of Lipid Rafts Clusters in db/db Mice

**DOI:** 10.1371/journal.pone.0106065

**Published:** 2014-08-28

**Authors:** Marta Pilarczyk, Lukasz Mateuszuk, Anna Rygula, Mariusz Kepczynski, Stefan Chlopicki, Malgorzata Baranska, Agnieszka Kaczor

**Affiliations:** 1 Faculty of Chemistry, Jagiellonian University, Krakow, Poland; 2 Jagiellonian Centre of Experimental Therapeutics, Krakow, Poland; 3 Department of Experimental Pharmacology, Jagiellonian University, Krakow, Poland; Cornell University, United States of America

## Abstract

Lipid rafts (LRs) are dynamic, sterol- and sphingolipid-enriched nanodomains involved in the regulation of cellular functions and signal transduction, that upon stimuli, *via* (e.g. association of raft proteins and lipids), may cluster into domains of submicron or micron scale. Up to date, however, lipid raft clusters were observed only under artificially promoted conditions and their formation *in vivo* has not been confirmed. Using non-destructive approach involving Raman and Atomic Force Microscopy imaging we demonstrated the presence of clustered lipid rafts in endothelium of the aorta of the db/db mice that represent a reliable murine model of type 2 diabetes. The raft clusters in the aorta of diabetic mice were shown to occupy a considerably larger (about 10-fold) area of endothelial cells surface as compared to the control. Observation of pathology-promoted LRs confirms that the cellular increase of lipid content results in clustering of LRs. Clustering of LRs leads to the formation of assemblies with diameters up to 3 micrometers and increased lipid character. This massive clustering of lipid rafts in diabetes may trigger a signaling cascade leading to vascular inflammation.

## Introduction

In 1987, van Meer *et al.* [1] using 7-nitrobenz-2-oxa-1,3-diazole (NBD)-labeled probes quantified fluorescent lipids sorting on the apical and basolateral sides of epithelial cells. They found that NBD-glucosylceramide was four-fold enriched on the apical membrane contrarily to NBD-sphingomyelin that was equally distributed both on the apical and basolateral sides [1]. This experiment has created the idea of lipid rafts (LRs), i.e. microdomains with properties different from the surrounding cell membrane. In 1992, Brown and Rose [2] discovered that glycosylphosphatidylinositol(GPI)-anchored proteins and glycosphingolipids are enriched in detergent-resistant membranes (DRMs) isolated in cold detergent extraction, whereas phospholipids are depleted from DRMs. This finding initiated an enormous growth of studies on these lipid-rich structures; it is enough to say that the Scopus search returned over 2000 citations with “lipid raft” in the article title! Nevertheless, up to now there is no apparent agreement on the size, composition, and function of LRs [3]–[8]. Moreover, the conclusions obtained by studying model membrane systems are difficult to verify in real cell systems, mainly due to lack of methods enabling measurements of transient nano objects. [3], [7], Nevertheless, today it is quite well established experimentally that the self-organization of proteins and lipids regulates the bioactivity of cell membranes [9]. The up-to-date definition of membrane rafts describes them as “small (10–200 nm) heterogeneous, highly dynamic, sterol- and sphingolipid-enriched domains that compartmentalize cellular processes. To form larger platforms small rafts can sometimes be stabilized through protein-protein and protein-lipid interactions [5]. In fact, the size of the observed domains varies between 4 nm (lipid shell size [6]) to several micrometers in model systems [10]. The size of LRs makes them ungrateful subjects for *in vivo* studies, although there are several experimental evidences of local clustering of protein and lipid reporters of various size [6], observed also directly in the nanoscale in living cells [11]. Other evidences for nanoscale clustering were gathered with new microscopic techniques, as reviewed in the recent work by Simons and Gerl [12]. The total internal reflection fluorescence (TIRF) microscopy and single quantum dot tracking were used to study the level of cholesterol in lipid rafts, while the protein behaviour was investigated by fluorescence correlation spectroscopy (FCS) [13]. Additionally, super-resolution optical microscopy methods e.g. stimulated emission depletion (STED) [14], stochastic optical reconstruction microscopy (STORM) [15], and scanning near optical microscopy (SNOM) [16] can were used to reveal the lipid – protein correlation in the plasma membrane. The majority of the methods used to study lipid rafts is based on fluorescence and/or require an initiator for cross-linking, which results in clustering of these dynamic, transient nanostructures into more stable, resting, microdomains [12]. For instance, the cross-linking of proteins ganglioside (GM1) and caveolin-1 (CAV-1) with cholera toxin (CTX) and anti-CAV-1 antibody, respectively, results in raft clustering to the size observable under an optical microscope. Nevertheless, such observations are inherently perturbed by the marker conjugation, i.e. the antibody cross-linking itself induces raft clustering by recruitment of suitable membrane constituents [12].

Caveolae are considered as a specific type of membrane rafts of a characteristic flask shape and various functions related mostly to signal transduction and lipids’ regulation [17], [18]. Caveolae are expressed in various cells, particularly in smooth muscle cells, fibroblasts, adipocytes, and endothelial cells. In the latter they constitute more than one-third of the overall surface of the plasma membrane [17], in agreement with a high level of CAV-1 expression in these cells [19].

Crucial cellular functions of rafts, including signal transduction, protein sorting, and synaptic transmission, were shown to be related to cholesterol and/or sphingolipids depletion [4]. It was suggested that the cholesterol content may activate inflammatory signaling pathway and contribute to the development of atherogenesis [20], closely related to type 2 diabetes mellitus (T2DM). In endothelial cells, endothelial nitric oxide synthase (eNOS) is targeted by LRs and caveolae of the plasma membrane and Golgi apparatus [19]. Expression of CAV-1 leads to inhibition of the NO release, i.e. binding caveolin-1 keeps eNOS in an inactive state [21]. CAV-1 is also related to the insulin secretion and insulin mediated signaling. [21] In this light, it is not surprising that lipid rafts may play a key role in the early onset and development of T2DM, as well as, may be involved in the regulation of the endothelium phenotype.

In this work we present LRs clustering in T2DM visualized by application of Raman and atomic force microscopy (AFM) imaging. Raman spectroscopy is a label-free technique with great potential for providing information about the biochemical composition of samples. Generally, pathological anomalies lead to chemical changes, which affect the vibrational spectra. Previously, Raman spectroscopy has been widely used to investigate pathological tissues. [22]–[25] The chemical specificity of Raman imaging enabled recognition of non-externally-induced LR assemblies in the vascular wall endothelium and their chemical characterization. Parallel AFM imaging of tissue topography and phase resulted in the determination of the exact size of these structures and enabled to link this information with the LR chemical composition. Immunohistochemical staining with anti-CAV-1 antibody confirmed the identity of the observed structures. The visualization of pathology-induced clustering of LRs opens a new perspective for studying the role and formation mechanisms of these assemblies in T2DM and others endothelium-related pathologies.

## Materials and Methods

The methodology based on simultaneous use of Raman spectroscopy and AFM was applied for imaging of the same area of the sample to gain information about both chemical composition and physical properties of the studied area. A Raman microscopic image is obtained by parallel measurement of spectral and spatial information, where the spectral information is the result of inelastic (Raman) scattering of the incident light illuminating the sample [26]. A microscope is used to transfer both the incident (laser) light and the Raman scattered light, therefore, the obtained information is derived from a very small volume of the sample (voxel) limited by the resolution of the optical microscope. Raman images are recorded by scanning the sample in the x and y directions and acquiring a complete Raman spectrum at each voxel. Therefore, Raman imaging results in a set of Raman spectra containing chemical information about the sample. Finally, the intensity of the “marker” (characteristic for a given component) band is calculated in each Raman spectrum and, after color-coding, it becomes a visual representation of the component distribution in the sample.

Atomic Force Microscopy (AFM) imaging is based on scanning the sample surface with the tip mounted at the end of the optically controlled, flexible cantilever [27]. Several different modes of operation are commonly used e.g. the tapping (AC) mode applied to soft samples such as cells and tissues. In tapping mode [28], [29] the tip is oscillating with the close to resonance frequency and high amplitude when the tip is under non-contact conditions. When the oscillating tip approaches the sample surface, the amplitude of the vibration is damped, therefore the oscillation amplitude can serve as a feedback parameter to measure the surface topography. In addition to the topography, the AC mode enables registration of other wave-related properties including a phase image, visualizing compressibility and/or hydrophobicity of the sample surface [30].

Application of combined Raman and AFM imaging to study the same sample area results in gaining information about the chemical structure of the sample along with its surface characterization (topography, stiffness, compressibility etc.). This methodology has previously been used to study murine tissue. [31].

The db/db mice represent a genetically-modified model (leptin receptor–deficient mice) of type 2 diabetes [32]. The samples were resected from a thoracic fragment of the aorta taken from the db+ (heterozygotic) or db/db (homozygotic) mice at the age of 16 and 20 weeks. The resected and split-open arteries were tightly glued to the cell-Tak-coated calcium fluoride surface (*en face* preparation). Subsequently, the tissue was preserved by a ten-minute soaking in formalin and rinsing twice with distilled water. Overall, the four 16-weeks old (2 db+ and 2 db/db, respectively) and three 20-weeks old (1 db+ and 2 db/db, respectively) mice were studied.

Raman imaging and AFM analysis was done with a Confocal Raman Imaging system WITec alpha 300 using 100×air objective (Olympus, MPlan FL N, NA = 0.9). The laser excitation wavelength of 532 nm, laser power of *ca.* 5–10 mW and the integration time of 0.2 second per spectrum were used in all cases. Images of an edge length 15×15 µm (75×75 pixels, one scan per point, 5652 spectra in total) or 4×4 µm, in the case of depth profiling (40×40 pixels, one scan per point, 1600 spectra in total), were acquired. In each case, the number of measured voxels was at least three-fold the edge size in order to satisfy the Nyquist criterion. The spectral resolution was 3 cm^−1^.

AFM measurements were performed in the AC mode with the Force Modulation probes (k = 2.8 N/m, WITec). The resolution of images was 200×200 pixels for the area of 20×20 µm.

Depth profiling of the tissue was obtained by multiple imaging of the same area in several layers of the sample with a 0.4–1 µm step in the z-direction. The layers marked as z = 0.0 µm were chosen by maximizing the intensity of the oscilloscope signal.

Data matrices were analyzed using a WITec Project software (background subtraction using a polynomial of degree 2 and the automatic removal of cosmic rays). The analysis of the spectra was supported by a Cluster Analysis (CA) (K-means, Manhattan distance, WITec Project Plus). CA was applied routinely to analyze all the hyperspectral imaging data. A ImageJ processing program [Rasband, W. S.; U. S. National Institutes of Health: Bethesda, Maryland, USA, http://imagej.nih.gov/ij/, 1997–2012.] was applied to calculate the LRs areas.

For immunohistochemical staining, each tissue was surrounded by a hydrophobic marker (Novocastra) and washed with PBS. In the first step, endogenous mouse antibodies were blocked using the MOM blocking reagent (VectorLabs) to reduce the background. Then preincubation with 5% normal goat serum (JacksonImmuno) and 2% non-fat dry milk in PBS for 30 min were used to reduce non-specific binding. For fluorescent detection of endothelial caveolin-1 and lipid raft formation, the sections were incubated in humid chambers with polyclonal rabbit anti-CAV1 antibody (Sigma) and FITC- conjugated cholera toxin B subunit (Sigma), respectively. After rinsing in PBS, the secondary biotinylated goat anti rabbit Ig was applied (JacksonImmuno). Following another rinse in PBS, the sections were incubated with Cy3- conjugated streptavidin (JacksonImmuno) to visualize the primary antibody binding site. Finally, nuclei were stained using a Hoechst 33258 solution (Sigma).

3D fluorescence images of immunostained lipid rafts were acquired with an A1-Si Nikon (Japan) confocal laser scanning system built onto a Nikon inverted microscope Ti-E using a Plan Apo 100×/1.4 Oil DIC objective. The images were acquired at a resolution of 1024×1024. Specimens were excited with 405, 488, and 561 nm diode lasers. 3D pictures were reconstructed using NIS-Elements AR 3.2 software.

### Ethics Statement

Dr. Lukasz Mateuszuk (Jagiellonian Centre for Experimental Therapeutics) was granted a formal waiver of ethical approval for animal work. The agreement was made by II local ethical committee for the animal experiments from Institute of Pharmacology Polish Academy of Sciences in Krakow number 955/2012 in 26^th^ July 2012. This agreement is valid for 3 years.

All efforts were made to minimize suffering. Mice were anesthetized with ketamine (Vetoquinol) and xylazine (Bayer) solution in doses of 80 mg and 8 mg per kg body weight, respectively. The drugs were administered intraperitoneally.

## Results and Discussion

### Chemical characteristics of lipid rafts

**Figure 1:**
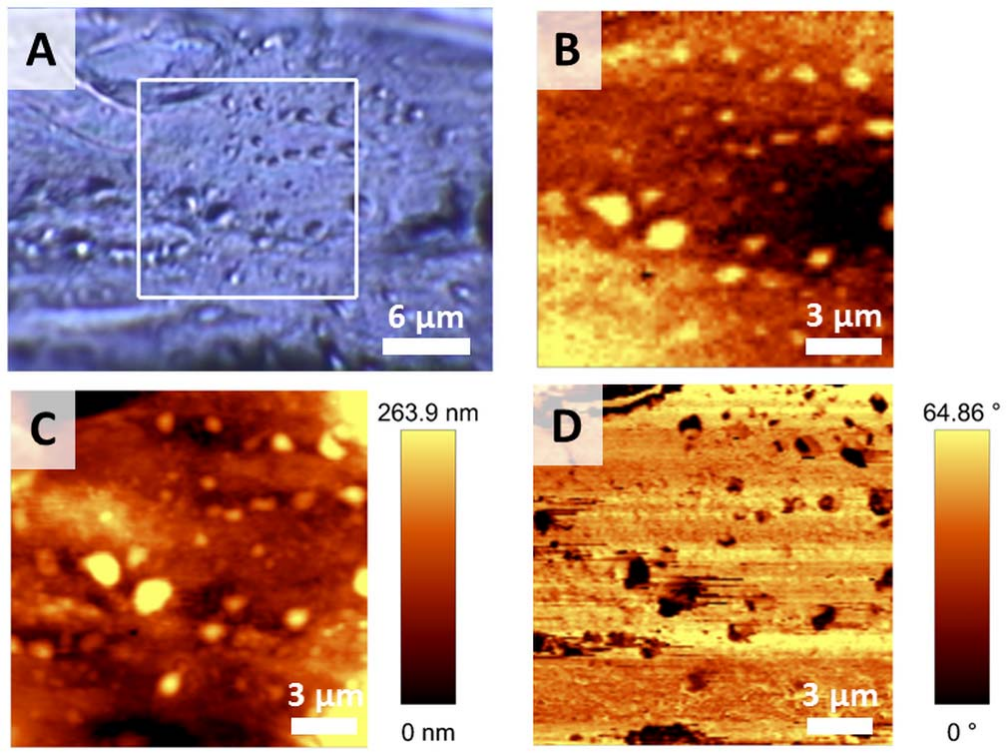
Representative visual, Raman and AFM images of the *en face* db/db vascular wall. The microphotograph of a studied tissue (100x, **A**), the Raman distribution image obtained by integration of the band in the 2800–3100 cm^−1^ range (**B**) and the complementary topography (**C**) and phase (**D**) AFM images.

Raman imaging is an emerging tool in medical diagnostics and has been already applied in the study of various pathologies [22]–[25], [33]–[35]. The db/db mice represent a genetically-modified model (leptin receptor–deficient mice) of T2DM [32]. The representative Raman distribution image of the *en face* (split-open) vascular wall of a db/db mouse together with the AFM topography and phase images of the studied surface are shown in Fig. 1 (compare Fig. S1, Supporting Information for the control).

Spherical structures with sizes up to 3 µm (Fig. 1) were identified in the studied tissues of db/db mice and assigned to aggregated LRs. In the Raman distribution images, these structures were distinguished, among others, by the very strong intensity of the C-H stretching band in the 2800–3100 cm^−1^ range (Fig. 1B) related to all organic components in the sample (mostly proteins and lipids). In the AFM images these assemblies were observed as protuberances of the tissue with a diameter between 300–3000 nm and a height/depth of 30–300 nm (Fig. 1C, topography) and significantly different physicochemical properties from the surrounding tissue (Fig. 1D, phase). The increased intensity of the ν_C-H_ Raman band for LRs corresponds to the increased tightness of packing due to the higher degree of order in LRs relatively to the surrounding tissue [36] and directly correlates with considerably different compressibility of these areas shown in the AFM phase images. The combined topography and phase images clearly demonstrate that these structures are localized on the uppermost layer of the endothelium over the plasma membrane. The topography indicates the existence of the protuberances on the very top of the tissue, while the phase image confirms that the lipid-rich structures have different properties than the rest of the tissue (and excludes the probability of structures localized just below the plasma membrane, for instance lipid droplets protruding above the tissue and covered by the membrane).

**Figure 2:**
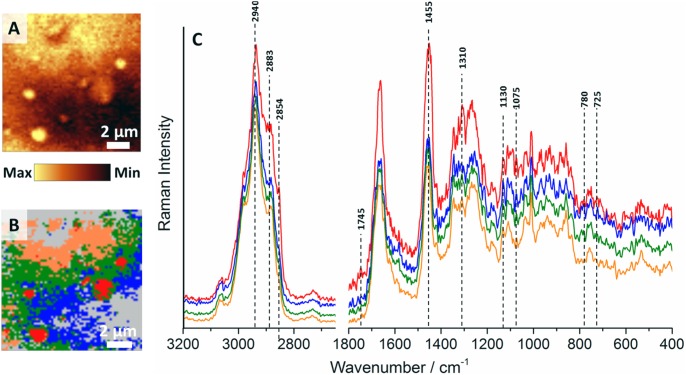
Representative Cluster Analysis of the *en face* db/db vascular wall sample. The Raman distribution image of the db/db sample obtained by integration of the band in the 2800–3100 cm^−1^ range (**A**), the CA results (K-means, Manhattan distance, 5 classes; **B**) and the complementary average spectra of classes (spectra were normalized to the 1011 cm^−1^ band and offset in order to emphasize the differences between them, the Raman intensity in the 2700–3200 cm^−1^ range is 3-fold magnified relatively to the fingerprint region, **C**).

Chemically, rafts are lipid-protein structures with raft proteins containing at least one transmembrane domain or hydrophobic modification [37] such as GPI-anchor [38], double acylation [10] or palmitoyl group [39] and rich in saturated sphingolipids and phospholipids [8]. To extract information about the chemical structure of raft assemblies, CA (K-means, Manhattan distance) was applied in order to isolate them from other components of the sample and characterize their spectral signature in detail. Fig. 2 presents the representative CA result of a Raman image of the *en face* db/db sample.

**Figure 3:**
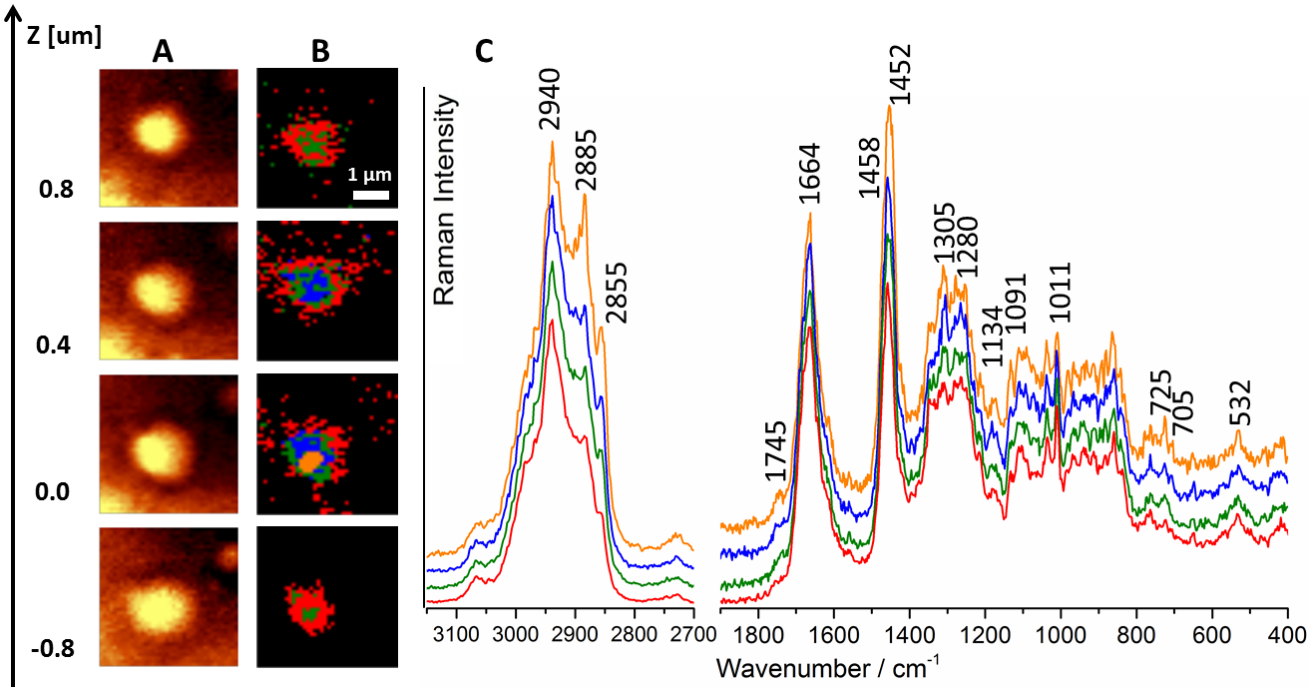
Depth profiling of the lipid raft. Raman distribution images (integration over the band in the 2800–3100 cm^−1^ range) at different depths (denoted by z value, the layer marked as z = 0.0 µm was chosen by maximizing the Raman intensity signal, **A**), Cluster Analysis of the corresponding images (**B**) with the averaged spectra of classes (**C**, spectra were normalized to the 1011 cm^−1^ band and offset in order to emphasize the differences between them, the Raman intensity in the 2700–3200 cm^−1^ range is 3.5-fold magnified relatively to the fingerprint region).

LRs, marked in red color in the cluster map (Fig. 2B) have a considerably different average spectrum (red line, Fig. 2C) than other classes identified in the tissue. Bands that appeared or increased in intensity in the fingerprint region of the average raft spectrum were assigned mostly to the lipid-type components. The band at 1745 cm^−1^ was attributed to the carbonyl stretching vibrations in esters, both phospholipids and triglycerides [24]. Features observed at 1455–1446 cm^−1^ with a significantly higher intensity in the raft spectrum were related to lipids in general (the CH_2_ bending vibration mode) and bands in the 1300–1315 cm^−1^ range are were attributed to the CH_2_/CH_3_ twisting vibrations. Bands at 1130 and 1075 cm^−1^ are were assigned to the C-C stretching vibrations of acyl chains [24]. The feature at 1099 cm^−1^ was due to the symmetric phosphate stretching mode [24]. The very characteristic band at *ca.* 725 cm^−1^ (Fig. 2 and 3) was related to the symmetric stretching vibrations of the N^+^(CH_3_)_3_ choline group (718 cm^−1^ in phosphatidylcholine and sphingomyelin, and 721 cm^−1^ in phosphatidylinositol, respectively) [40]. The latter assignment was confirmed by the presence of the feature at *ca.* 780 cm^−1^, attributed to the O-P-O bending mode, and observed at the similar wavenumber in the spectrum of phosphatidylinositol (776 cm^−1^) [40]. Additionally, the C-H stretching region considerably differentiated the raft and non-raft spectra. The hallmark of rafts was a considerable increase of the components at *ca*. 2854 and 2883 cm^−1^ relative to the one at 2940 cm^−1^ (resulting in an overall increase of the ν_C-H_ band integral intensity). It is rationalized by the increase of lipid to protein ratio in rafts compared to other areas of the tissue (features at 2854, 2883 and 2940 cm^−1^ can be assigned to ν_s_(CH_2_) in lipids and fatty acids, ν_s_(CH_3_) in lipids and fatty acids and ν(C-H) in lipids and proteins, respectively [24]).

In general, the obtained average raft spectra unequivocally showed the presence of the lipid-protein structures. The spectra were dominated by features due to fatty acids with the phosphocholine group, most probably sphingomyelin. The presence of sphingomyelin was highly expected as it is a major lipid component of caveole-related domains [41]. Moreover, the apparent heterogeneity in the raft composition inside the single raft assembly was observed upon depth profiling of the *en face* sample (Fig. 3; compare also Fig. S2, Supporting Information).

Up to four classes were extracted for the images recorded at different depths of the representative raft, showing a clear difference in the composition of the lipids/proteins between these layers. For instance, the features at 2885 and 705 cm^−1^, very characteristic for cholesterol and cholesterol esters [24], were more pronounced in the average spectrum of the orange class. Additionally, the features, characteristic to phosphatidylinositol, appeared in the orange spectrum, namely bands at 778 and 413 cm^−1^, along with the increased intensity of the bands at *ca.* 725 cm^−1^ (sphingolipids) [40]. Overall, all the layers showed the sphingolipid-protein signature, while the inner layer (the orange class) was richer in phosphatidylinositol-type compounds and cholesterol esters. As it will be shown below, raft composition varied also depending on the size of these structures.

### Lipid rafts: db/db versus control

**Figure 4:**
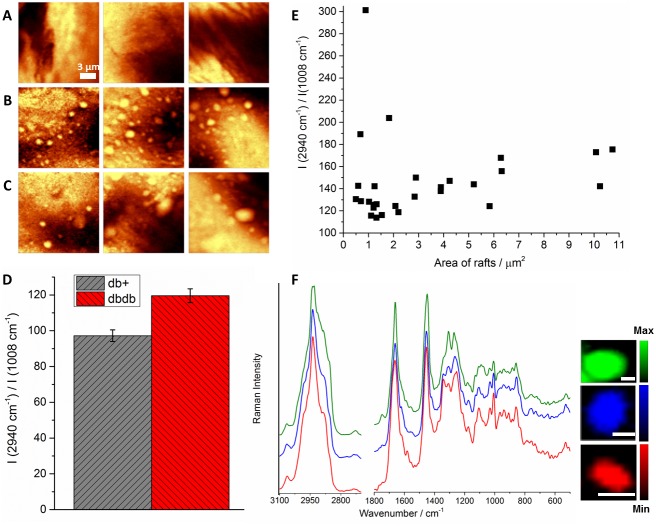
Clustering of lipid rafts in T2DM. Comparison of the representative Raman distribution images (integration over the band in the 2800–3100 cm^−1^ range) of the *en face* endothelium of db+ (control, **A**), 16-weeks-old db/db (**B**) and 20-weeks-old db/db (**C**) mice. The overall endothelium lipid content (defined as the average intensity ratio of the band at 2940 to the band at 1008 cm^−1^ in all measured db/db or db+ samples; **D**). The individual lipid content of LRs (defined as the intensity ratio of the band at 2940 to the band at 1008 cm^−1^ for individual LR assemblies) as a function of their area (**E**). The correlation between the average spectra of representative LRs and their size (the scale bar denotes 1 µm, the spectra were normalized to the 1011 cm^−1^ band and offset in order to emphasize the differences between them, the Raman intensity in the 2700–3200 cm^−1^ range is 3-fold magnified relatively to the fingerprint region; **F**).

The relationship between LRs/caveolae and eNOS [17], [19], [21] and decreased NO level in T2DM raise the question whether the formation of lipid rafts is affected by the development of this pathology. The comparison of the Raman images of the *en face* samples of db+ (control, Fig. 4A) and db/db (T2DM, Fig. 4B, C) mice showed striking differences between the tissue area covered by LRs in T2DM relatively compared to the control.

Sparse lipid-rich assemblies were found in the db+ tissues, while the db/db fragments contained numerous protuberances and invaginations on the very top surface of the tissue regardless of the age of the studied mice (16 or 20 weeks old). Overall, there was about a ten-fold increase in the endothelial surface covered by lipid-rich platforms in T2DM compared to the control for both 16 and 20 weeks old diabetic animals (Fig. S3, Supporting Information). The clustering of lipid rafts had a considerable impact on the average Raman spectra, as it causes an increase in the overall lipid content in the endothelium. The overall lipid content can be statistically measured as the ratio of the integral intensity of the band for lipids and proteins (centered at 2940 cm^−1^, assigned to the C-H stretching vibrations) to the well-defined protein marker band (at *ca.* 1008 cm^−1^, attributed to the ring breathing mode of phenylalanine) in the obtained Raman images (Fig. 4D). It is clear that the overall lipid content increased in T2DM compared to the control (Fig. S4, Supporting Information). Moreover, the lipid-to-protein ratio in the individual LRs increased with increasing size of LRs (Fig. 4E), which was shown in the average raft spectra as increase of bands due to lipids in bigger assemblies relative to smaller rafts (Fig. 4F). After rejecting three outliers (N = 25, 5% level of decision), the p-value equals 0.0025 (Fig. S5, Supporting Information), showing that the correlation between the lipid content and the raft area was significant and that the lipid-to-protein ratio increased with increasing of the raft area.

### Lipid rafts: aggregation versus T2DM-progression

**Figure 5:**
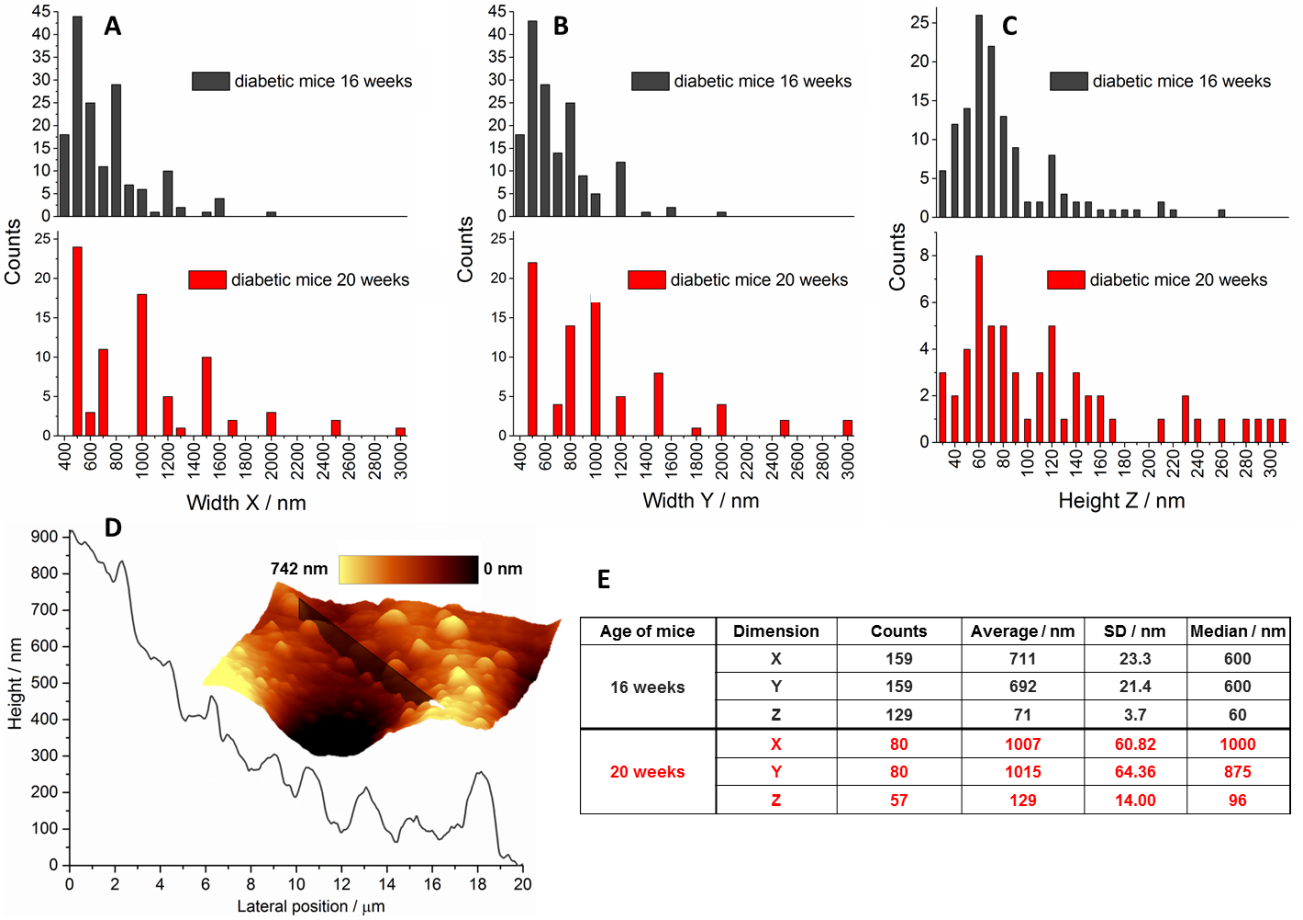
Size statistics of clustered lipid rafts in T2DM. The x (**A**), y (**B**) and z (**C**) dimensions of LRs observed for 16-weeks-old (black) and 20-weeks-old (red) mice determined based on the acquired AFM topography images (the example in **D**: a representative topography image along with a topography cross-section) and the summary of statistical data related to the LRs size (**E**).

To account for the shape and exact size of clustered LRs, the x, y and z dimensions of individual lipid-rich structures were read off the AMF topography images and compared below (Fig. 5).

The results presented in Fig. 5 indicate that the shape of rafts observed in our experiment resembles an ellipsoid of similar x and y widths, and of a significantly smaller height (x≅y≥z). LRs size spans the range of 300–3000 nm in x, y and 30–300 nm in z (Fig. 5A–C). Taking into account a very good vertical resolution of AFM (*ca.* 1 nm) and the typical height of the lipid bilayer (a few nanometers) it was concluded that the clustered LRs, observed in pathological conditions, considerably overgrew the cell membrane. The AFM images also strongly confirmed that the observed raft assemblies were located in the uppermost layer of the endothelium. The size of LRs is considerably age-dependent. With the development of T2DM both the diameter and, particularly, the height of LRs increased (Fig. 5E), due to the progressive clustering of the raft assemblies into larger platforms, up to 3 micrometers in diameter for 20-weeks old animals.

**Figure 6:**
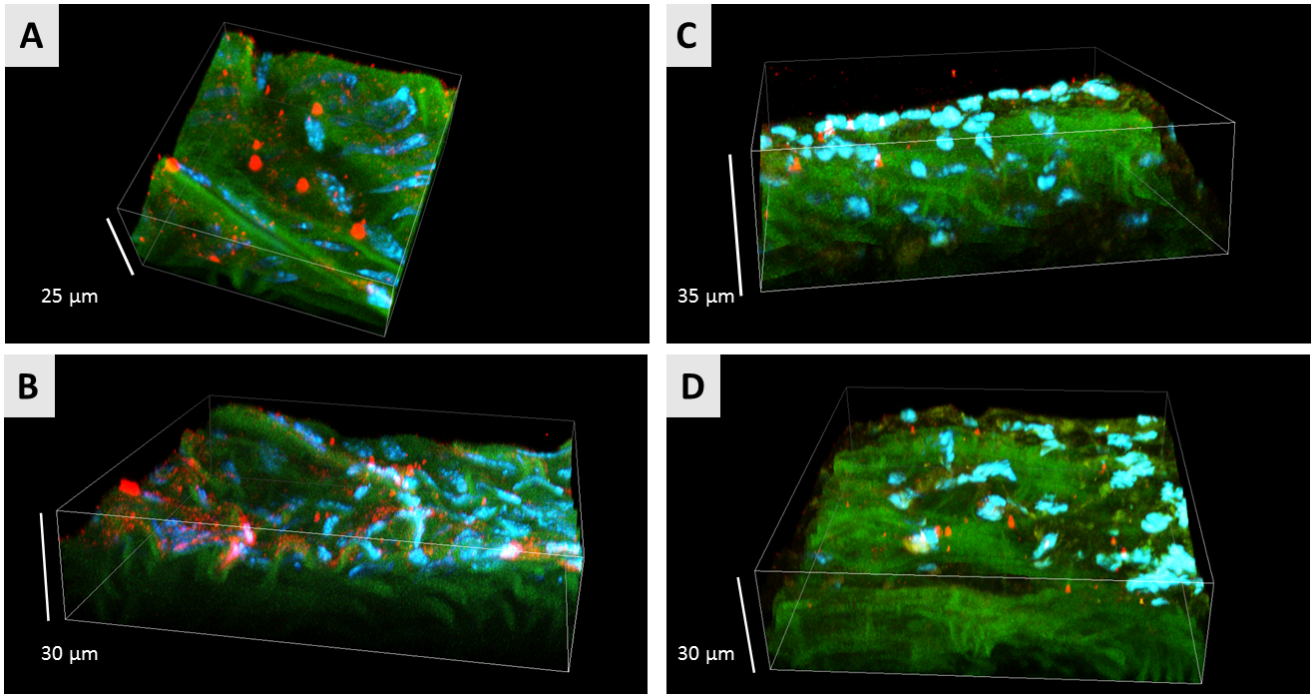
Representative images of lipid rafts obtained using confocal laser scanning microscopy. Two representative confocal micrographs of db/db (**A, B**) and db+ (**C, D**) tissue fragments. Endothelial caveolin-1, cell nuclei and elastin fibers are visualized in red, blue and green channels, respectively.

Immunohistochemical staining with anti-CAV-1 antibody confirmed the existence of CAV-1 rich patches in the studied tissues (Fig. 6).

Although the Raman/AFM and fluorescence images are not directly comparable due to the possibility of additional co-clustering of LRs upon the introduction of the external probe, the general picture inferred from the fluorescence images confirmed the Raman and AFM findings. Particularly, (i) significant proliferation of LRs in the endothelium of db/db mice versus the control db+ subjects and (ii) association of these structures into larger assemblies with the progress of the pathology, are clearly observed.

Micrometer-size raft phase has previously been detected in model systems [12]. Our *ex vivo* study of aggregated LRs confirmed the previous findings obtained in membrane models. We showed clearly that also in the tissue the increase of the overall lipid content, associated with the pathology progress, results in a dramatic increase in the LRs aggregation in the endothelium. Thus, a hyperlipidemia associated with the T2DM development resulted in promoting of an overall increase of the lipid content followed by clustering of LRs into larger assemblies and size-dependent increased lipid character. The significant proliferation of LRs in T2DM explained NO deficiency occurring likely due to inhibition of eNOS activity [21]. Increased levels of saturated fatty acids, associated with diabetic hypertriglyceridemia [42], were suggested to increase recruitments of toll-like receptors (TLRs) into lipid rafts [43]. TLRs are responsible for activation of the immune cell responses and their stimulation *via* saturated fatty acids, which is associated with the alteration of the cell membrane properties [42]. Additionally, it was observed that the reduced levels of serine palmitoyltransferaze, the first enzyme in *de novo* biosynthetic pathway of sphingomyelin, may decrease both the sphingomyelin levels and the function of lipid-raft associated proteins, for example TLR4 [44]. Overall, the observed LRs clustering in T2DM was involved with the increased signal transduction and changed the immune response of the vascular endothelium in the pathological state. Our approach, enabling non-induced visualization of clustered LRs opens a new perspective for LRs study in *in vitro* and *in vivo* samples.

## Conclusions

Clustered lipid rafts were observed on the surface of endothelium of the aorta of diabetic db/db mice. According to our knowledge, this is the first report showing the formation of these structures solely upon the pathology development and not artificially upon binding to any externally introduced fluorescent probes for instance cholera toxin.

Identification of the observed structures as lipid rafts was based on several experimental evidences of both chemical and biochemical origin. These structures were enriched in lipids as was clearly demonstrated by the Raman spectra. Assignment of sphingolipids as a component of these lipids was rather straightforward, as a few of functional groups in biological compounds show bands in the 700–750 cm^−1^ range. The bands due to sphingolipids were complemented with bands characteristic for cholesterol and phospholipids (Fig. 3), altogether accounting for all lipid constituents of rafts. The clear indication of a significantly different nature of the observed lipid patches were provided by the AFM phase images, confirming that these structures had considerably dissimilar character compared to the other areas of the tissue that can be related to increased order of raft structures. Additionally, the AFM topography images clearly demonstrated that the observed lipid structures were upper-most perturbations of the tissue. Finally, the presence of lipid rafts in the endothelium of db/db mice was independently confirmed by immunohistochemical staining showing the presence of caveolin 1, the protein characteristic only for lipids rafts.

Most of the observed structures had the diameter of ca. 500–600 nm and the height of ca. 60 nm, indicating that in the endothelium of db/db mice the rafts clustered into larger domains. There are growing evidences that the clustering of lipids rafts can occur on both sides of the membrane [8]. Antibodies, antigens, and raft-lipid-binding proteins can extracellularly link to LRs, while raft-clustering proteins have tendency to cluster on the intracellular side. Such “vertical” clustering undoubtedly increases the height of lipid rafts aggregates and justifies obtained heights of LRs in endothelium of diabetic animals. Finally, our conclusion about LRs was strongly supported by a comparison of the results obtained for the control and diseased (diabetic) mice. In the control animals, the number of aggregated LRs was scarce, while in the pathological conditions the area covered by these assemblies increased considerably. As the disease progressed, both the size and the structure of LRs changed. According to Simons and Gerl [12] nanoscale rafts assemblies in response to stimuli cluster into bigger structures that, in the model systems, further cluster into micrometer-sized raft phases. The observed changes upon the T2DM development reflected the findings in model systems. Our *ex vivo* study of non-induced, pathology-promoted LRs confirmed that the cellular increase of lipid content is associated with a clustering of LRs into larger assemblies with the diameter up to 3 micrometers and a systematic, size-related increase of their lipid character.

About ten-fold increase in the LRs area may be closely related to decrease of NO activity observed in T2DM probably due to eNOS inhibition by CAV-1 [21]. Thus, it might be that lipids raft clustering in the endothelium of diabetic mice is linked to perturbation of cellular homeostasis and may be involved in the triggering of signaling cascade leading to vascular inflammation.

## Supporting Information

Figure S1 **Representative visual, Raman and AFM images of the** ***en face*** **db+ vascular wall.** The microphotograph of a studied tissue (100x, **A**), the Raman distribution image obtained by integration of the band in the 2800–3100 cm^−1^ range (**B**) and the complementary topography (**C**) and phase (**D**) AFM images.
(TIF)

Figure S2 **Chemical heterogeneity of lipid rafts.** Comparison of average Raman spectra of four random lipid rafts (A) of the similar diameter along with Raman distribution images obtained by integration of the band in the 2800–3100 cm^−1^ range of a studied fragments of db/db samples (**B**, scale bar = 0.5 µm). Labels in **A** denote some characteristic bands due to lipids.
(TIF)

Figure S3 **Endothelium area covered by lipid rafts.** The Raman distribution image obtained by integration of the band in the 2800–3100 cm^−1^ range of a studied fragment of db/db sample (**A**), CA results (K-means, Manhattan distance) for a class assigned to lipid rafts (**B**), the area classified as lipid rafts counted in Image J processing program (Rasband, W.S., ImageJ, U. S. National Institutes of Health, Bethesda, Maryland, USA, http://imagej.nih.gov/ij/, 1997–2014), statistics related to calculated area of lipid rafts (**D**) with the visual representation (**E**).
(TIF)

Figure S4 **Average lipid content in tissues from db/db and db+ mice.** Integral intensity of marker bands due to lipids and proteins at 2940 and 1008 cm^−1^, respectively, in the individual samples along with the ratio of bands due to lipids and proteins (**A**), the average values for diabetic (db/db) and control mice (db+) with the standard deviation (**B**) and the overall endothelium lipid content (defined as the average intensity ratio of the band at 2940 to the band at 1008 cm^−1^ in all measured db/db or db+ samples; **C**).
(TIF)

Figure S5 **Statistical analysis of relationship between lipid-to-protein ratio** ***vs*** **area of rafts.** The raw data: area of rafts (calculated based on AFM images) and lipid-to-protein ratio (computed based on Raman images) (A) were used to create a plot (B). The statistics results (C): R Spearman and p-value was calculated with 5% level of decision for two data ranges: all data (red and black) and after rejection of three outliers (red). The Spearman's rank correlation coefficient was calculated because the Normality Test (Shapiro-Wilk) showed that the studied population cannot be considered a normal distribution population (with 5% level of decision). The obtained p-value equals 0.0025 (is considerably lower than 0.05) showing significant correlation and considerable increase of the lipid content with increasing area of lipids rafts.
(TIF)
