# Supplementary figures and images for: Endothelium in Spots – High-Content Imaging of Lipid Rafts Clusters in db/db Mice
Source: PLoS One. 2014 Aug 28;9(8):e106065. doi: 10.1371/journal.pone.0106065 (PMC4148353; doi:10.1371/journal.pone.0106065)

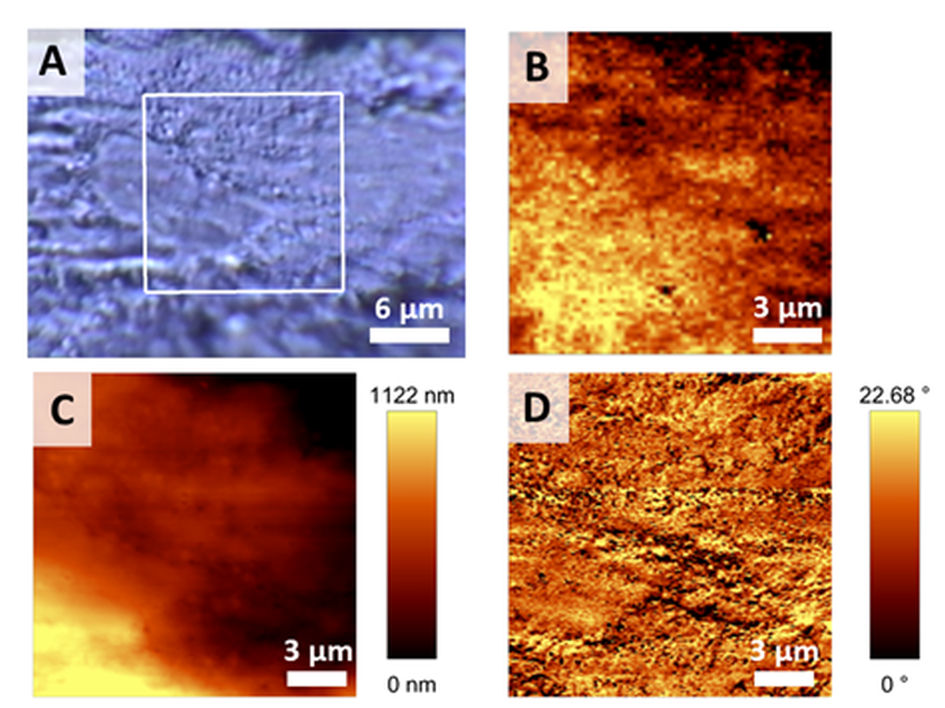

Supplement: Figure S1 — Representative visual, Raman and AFM images of the en face db+ vascular wall. The microphotograph of a studied tissue (100x, A), the Raman distribution image obtained by integration of the band in the 2800–3100 cm−1 range (B) and the complementary topography (C) and phase (D) AFM images. (TIF) [file pone.0106065.s001.tif]

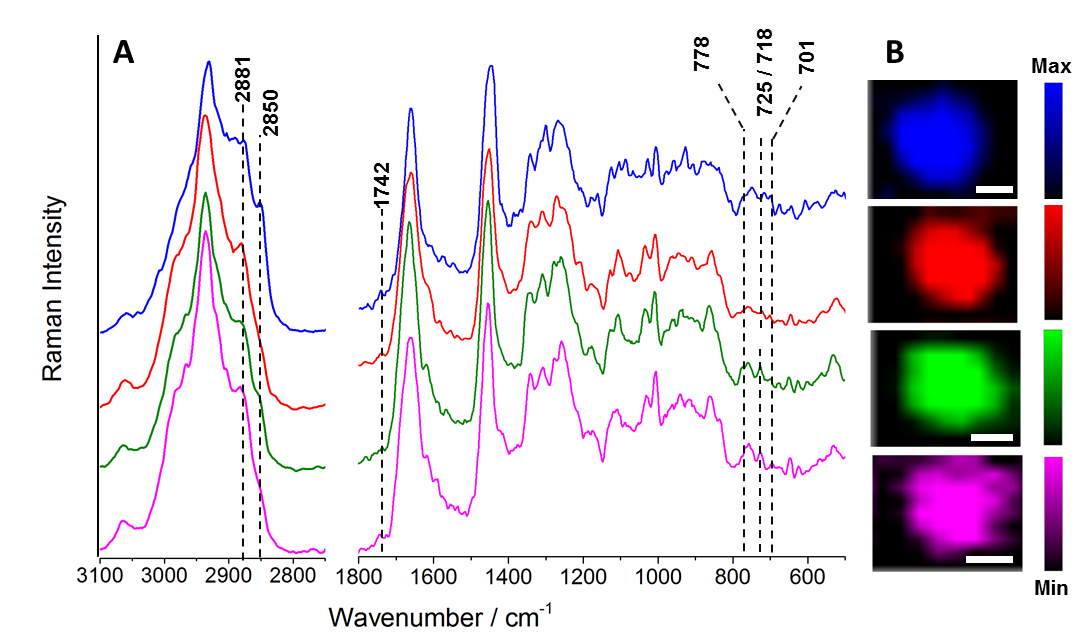

Supplement: Figure S2 — Chemical heterogeneity of lipid rafts. Comparison of average Raman spectra of four random lipid rafts (A) of the similar diameter along with Raman distribution images obtained by integration of the band in the 2800–3100 cm−1 range of a studied fragments of db/db samples (B, scale bar = 0.5 µm). Labels in A denote some characteristic bands due to lipids. (TIF) [file pone.0106065.s002.tif]

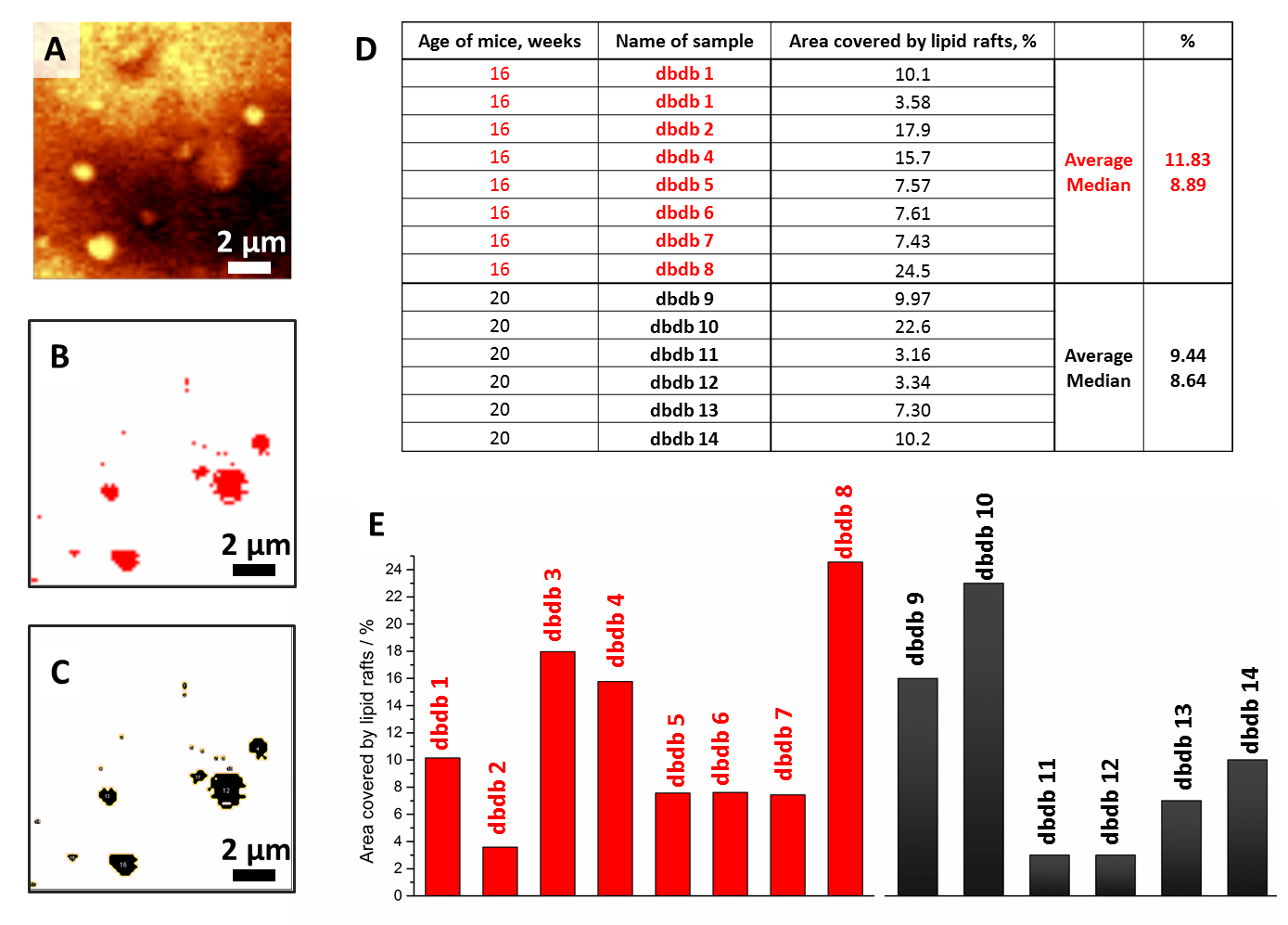

Supplement: Figure S3 — Endothelium area covered by lipid rafts. The Raman distribution image obtained by integration of the band in the 2800–3100 cm−1 range of a studied fragment of db/db sample (A), CA results (K-means, Manhattan distance) for a class assigned to lipid rafts (B), the area classified as lipid rafts counted in Image J processing program (Rasband, W.S., ImageJ, U. S. National Institutes of Health, Bethesda, Maryland, USA, http://imagej.nih.gov/ij/, 1997–2014), statistics related to calculated area of lipid rafts (D) with the visual representation (E). (TIF) [file pone.0106065.s003.tif]

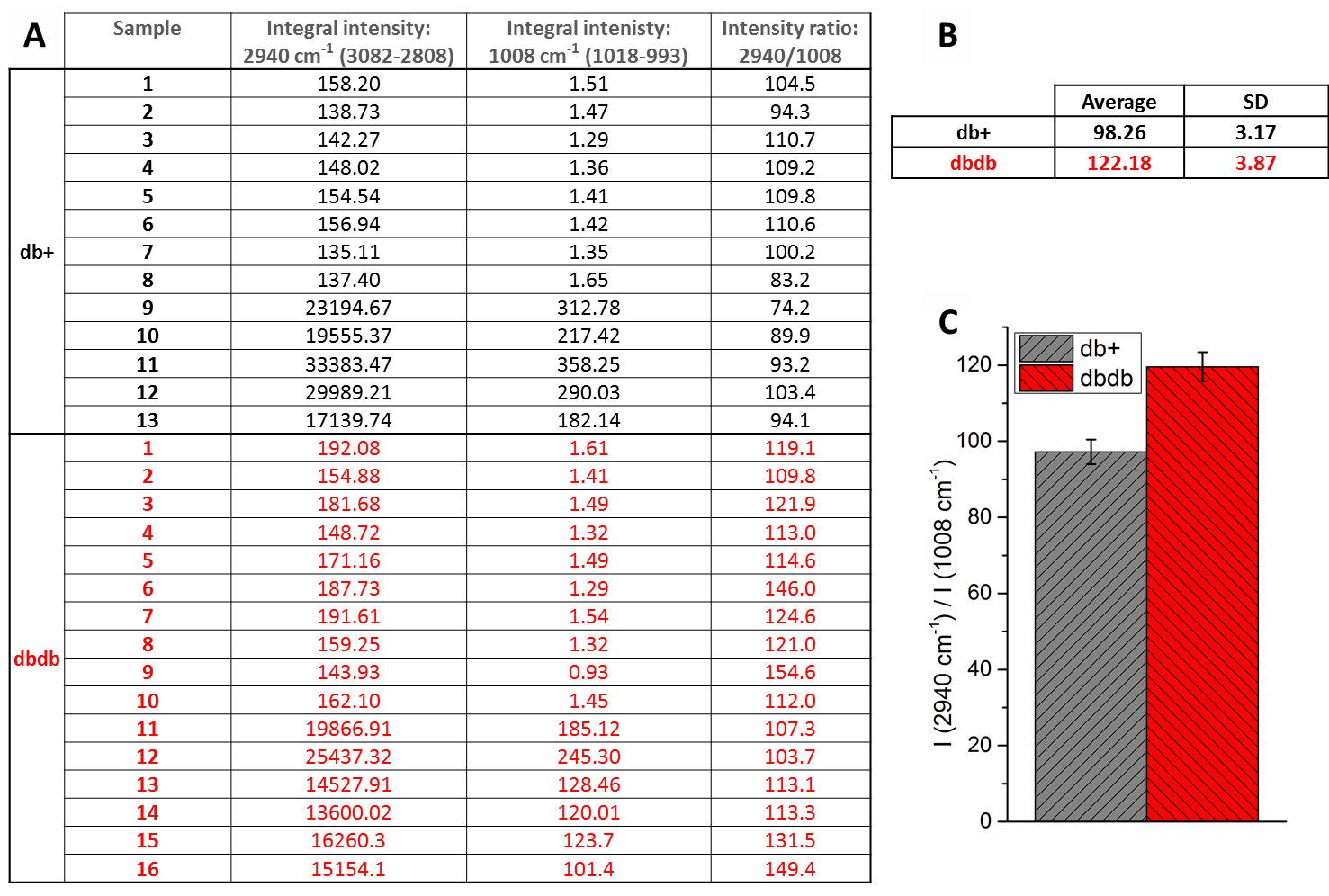

Supplement: Figure S4 — Average lipid content in tissues from db/db and db+ mice. Integral intensity of marker bands due to lipids and proteins at 2940 and 1008 cm−1, respectively, in the individual samples along with the ratio of bands due to lipids and proteins (A), the average values for diabetic (db/db) and control mice (db+) with the standard deviation (B) and the overall endothelium lipid content (defined as the average intensity ratio of the band at 2940 to the band at 1008 cm−1 in all measured db/db or db+ samples; C). (TIF) [file pone.0106065.s004.tif]

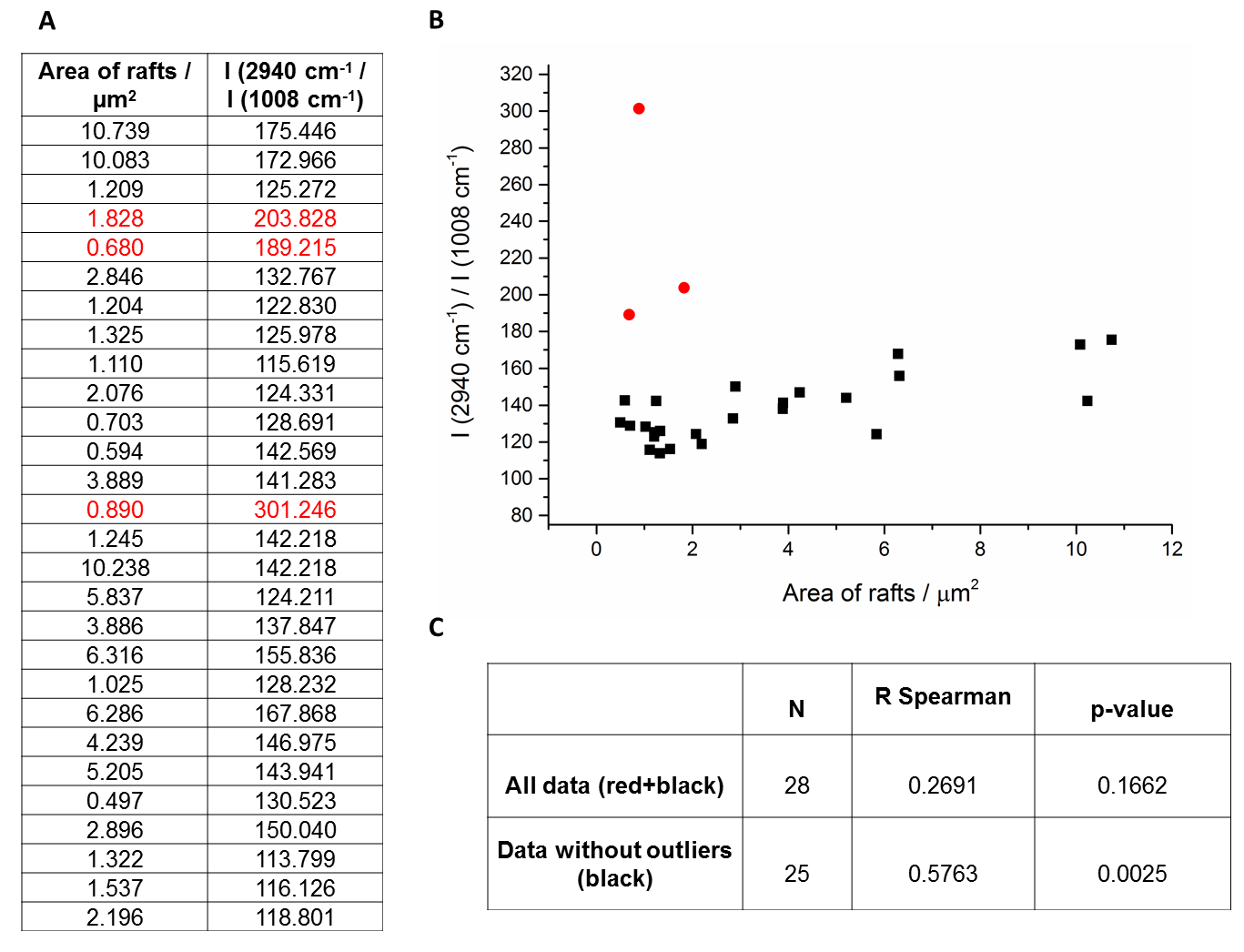

Supplement: Figure S5 — Statistical analysis of relationship between lipid-to-protein ratio vs area of rafts. The raw data: area of rafts (calculated based on AFM images) and lipid-to-protein ratio (computed based on Raman images) (A) were used to create a plot (B). The statistics results (C): R Spearman and p-value was calculated with 5% level of decision for two data ranges: all data (red and black) and after rejection of three outliers (red). The Spearman's rank correlation coefficient was calculated because the Normality Test (Shapiro-Wilk) showed that the studied population cannot be considered a normal distribution population (with 5% level of decision). The obtained p-value equals 0.0025 (is considerably lower than 0.05) showing significant correlation and considerable increase of the lipid content with increasing area of lipids rafts. (TIF) [file pone.0106065.s005.tif]
